# Support Needs, Barriers, and Facilitators for Fathers With Fear of Childbirth in Sweden: A Mixed-Method Study

**DOI:** 10.1177/15579883241272057

**Published:** 2024-09-13

**Authors:** Carita Nordin-Remberger, Margareta Johansson, Karin S. Lindelöf, Michael B. Wells

**Affiliations:** 1 Obstetric and Reproductive Health Research, Department of Women’s and Children’s Health, Akademiska University Hospital, Uppsala University, Uppsala, Sweden; 2 Women’s Mental Health During the Reproductive Lifespan—WOMHER, Uppsala University, Uppsala, Sweden; 3 Centre for Gender Research, Uppsala University, Uppsala, Sweden; 4 Department of Women’s and Children’s Health, Karolinska Institutet, Solna, Sweden

**Keywords:** barriers, facilitators, fathers, fear of childbirth, FOBS

## Abstract

The aim of this mixed-method study was to identify support needs, as well as barriers and facilitators to seeking support in a sample of Swedish fathers with a fear of childbirth (FOC). Participants completed an anonymous quantitative online survey (*N* = 131), with three free-text items for those self-identifying as having an FOC (*N* = 71) and five individual in-depth interviews. Data analysis included descriptive and chi-square analyses for quantitative data, and manifest content analysis for qualitative data. Those with a severe FOC were more likely to report having on-going mental health difficulties (*p* = .039) and one fifth (21%) of the participants with severe FOC wanted to receive professional treatment, but only 8.1% received treatment. Most participants either preferred individual support or to receive support together with their partner. Fathers with severe FOC were more likely to report one or more barriers than those without FOC (*p* = .005), where unwanted social stigma was the single largest barrier. Qualitative findings identified one main category: *Expectant fathers missing and wishing for support for FOC* composed four generic categories: (1) *support in developing an understanding of their fear*, (2) *coping by being aware of feelings*, (3) *professional support through trust and respect*, and (4) *needing individualized support*. To encourage healthy fathers, clinical professionals should find ways to support fathers, such as by providing them with their own perinatal appointments, asking them about their feelings, as well as screening, diagnosing, and treating fathers with severe FOC.

## Introduction

The World Health Organization promotes expectant fathers’ involvement in antenatal care (World Health Organization, 2007). However, for some men, the transition to fatherhood can negatively impact their mental health and well-being (Wong et al., 2016). As such, becoming a father incorporates feelings of both great expectations and joy, and fears and worries (Baldwin et al., 2018). Therefore, the transition can be difficult for some expectant fathers, where 13% have a severe fear of childbirth (FOC; Eriksson et al., 2005; Hildingsson, Johansson, et al., 2014), 5% to 10% have depression (Cameron et al., 2016; Paulson & Bazemore, 2010) and 3% to 25% have anxiety (Leach et al., 2016; Leiferman et al., 2021; Philpott et al., 2019). Expectant fathers with FOC report poorer physical and mental health than non-fearful expectant fathers (Hildingsson, Haines, et al., 2014), including greater levels of stress, anxiety, and depression (Leach et al., 2016; Philpott et al., 2017). In addition, expectant fathers’ FOC is further associated with expectant mothers’ anxiety and depression (Koh et al., 2015) and less satisfaction with the couple relationship (Cameron et al., 2021). These psychological distress factors can later affect children’s attachment patterns and emotional and cognitive development (Ramchandani et al., 2005). However, while pregnant women’s fears and worries are being acknowledged via receiving support (Dencker et al., 2019; Larsson et al., 2016; Lukasse et al., 2015; Lyberg et al., 2019; Nilsson et al., 2012; Rondung et al., 2016; Rouhe et al., 2011; Saisto et al., 2001), a significant knowledge gap exists in understanding the support needs, as well as the facilitators and barriers to expectant fathers’ seeking support for their FOC.

The prenatal period is a stressful time for some expectant fathers, including understanding their role during labor and birth (Steen et al., 2012), partner and baby health (Kowlessar et al., 2015), the changes to the couples’ relationship (Poh et al., 2014), concerns about childbirth and becoming a parent (Baldwin et al., 2018; Shorey & Chan, 2020), and accessing support for themselves (Venning et al., 2021). As such, many expectant fathers report feelings of uncertainty, helplessness, stress, fear, and anxiety (Philpott et al., 2019; Steen et al., 2012). These factors may undermine the necessary and important support they provide to their partners, and thus, potentially increase the risks of complications for expectant mothers and infants (Nylen et al., 2013).

There are several factors that are associated with FOC in expectant fathers. For example, fathers are more likely to have FOC if their partner also has FOC or if they have mental ill-health (Ghaffari et al., 2022). Other factors associated with expectant fathers developing FOC include having a low self-efficacy of childbirth and fatherhood, as well as displaying an inability to cope with their new life situation, negative thoughts regarding their partner’s pain during labor, including potential instrumental and surgical interventions, as well as concerns for their partner and the unborn baby’s health and well-being (Ghaffari, Sharif Nia, et al., 2021; Hanson et al., 2009).

## The Emotional Burden of Societal Expectations

w?>Societal gender constructs of expectant fathers suggests that they should remain strong, calm, and self-confident (Eriksson et al., 2006). In fact, in general, men are less likely to seek mental health support, and more likely to hold negative attitudes toward therapy, and to stop treatment compared with women (O’Brien et al., 2017). Expectant fathers may also feel like they cannot show or express their fearful emotions, as they do not want to worry their partner (Eriksson et al., 2005; Etheridge & Slade, 2017). As such, expectant fathers with FOC have a personal hidden burden leading to feelings of isolation, helplessness, crying, and being less involved in prenatal care and childbirth (Moran et al., 2021). Nearly, two thirds (65%) of expectant fathers with severe FOC reported that nobody, including clinical professionals, ever asked how they felt about childbirth (Eriksson et al., 2005).

Men are often socialized to suppress vulnerability and to project an image of strength and resilience, particularly in contexts traditionally associated with femininity, such as pregnancy and childbirth (Call & Shafer, 2015). In the context of FOC, these gender stereotypes may pose unique challenges for expectant fathers who experience anxiety or distress surrounding the childbirth process. Expectant fathers with FOC may feel pressure to conform to societal expectations of stoicism and bravery, leading them to suppress or minimize their fears and emotions (Call & Shafer, 2015; Primack et al., 2010). This may also prevent them from acknowledging their fears and accessing the support they need to cope with their fears.

Since an increased awareness of expectant fathers’ FOC needs, including understanding what support structures they want and can benefit from, can help reduce FOC, and thus improve expectant fathers’ perinatal mental health and overall well-being (Moran et al., 2021), further research is needed.

Using a mixed-method study design, the current study aims to assess (a) the extent to which fathers have a severe FOC, (b) to what extent they want to receive further support for their FOC and what kind of support they prefer and from whom, and (c) their barriers and facilitators to seeking clinical support, as well as support from family and friends.

## Materials and Methods

### Study Design

A cross-sectional concurrent mixed-method design, using an online anonymous survey, was used. In total, 131 participants completed the survey. Five fathers participated in a semi-structured interview via telephone. All data was collected between February and September 2022.

### Inclusion and Exclusion Criteria

Eligible participants were those: (1) self-identifying as a father (either expectant father or father of children), (2) 18 years of age or older, (3) living in Sweden, and (4) able to read and understand Swedish or English enough to complete an online survey. This study was approved by the regional ethical board in Sweden (No.: 2021-03759.)

### Sampling

Participants were recruited through convenience sampling via three recruitment strategies. The first recruitment strategy involved six hospitals across five counties in Sweden (Malmö, Karlstad, Stockholm, Uppsala, and Umeå). Potential participants were informed about the study via a midwife either at the end of an appointment or at the routine ultrasound. Potential participants were handed a study information brochure with a weblink and Quick Response (QR) code for the survey.

A second recruitment strategy involved advertising on social media (i.e., groups for fathers on Facebook and Instagram), on websites directed to fathers, and in the Män för jämställdhet (Men for Gender Equality), as well as Familjeliv (Family Life) newsletters, respectively. Both of these are non-profit organizations that support fathers. A weblink and QR code was posted in their respective newsletters, taking potential participants to the online survey.

A third recruitment strategy used paid Facebook advertisements via an advertising company in collaboration with Uppsala University. Paid Facebook advertisements were used for approximately 4 weeks (July–August 2022) to further recruit participants. At the bottom of the advertisement, there was a web link and/or QR code to the online survey.

In all three recruitment strategies, potential participants were redirected to a secure online data collection website (REDCap; Maré et al., 2022), where they were presented with an information letter and a consent form. Study information included that participation was voluntary, would not affect their received clinical care, and that answers could be anonymous also in relation to the research team, unless they volunteered their email address to participate in a future semi-structured interview exploring their experiences of FOC. Participants were advised that by clicking “yes” they consented to participate in the survey.

### Measurements

The survey comprised two parts: Part I consisted of questions on sociodemographic characteristics and obstetric items including the fear of birth scale (FOBS; 35 items). The last item in Part I asked if they experienced FOC right now. Response options to this item were on a 4-point Likert-type scale: (a) Not at all, (b) To a small extent, (c) To a large extent, and (d) To a very large extent. If the participant responded with any answer except “Not at all,” they were redirected to Part II of the survey. If the participant answered Not at all, the survey ended.

#### Sociodemographic Characteristics

Participants were asked their age (continuous), if they had previous children (Yes/No), civil status (married, living with someone, with a partner, but not living together, single), highest level of education (primary school/high school or equivalent/college/university), employment status (employed, studying, parental leave, sick leave, sickness compensation and unable to work, unemployed), country of birth (Sweden/Other Nordic countries/Europe/ Africa/Asia/Middle East/North America/South America/Oceania/Other), place of residence (city, town, village), partner pregnant (yes/no), planned pregnancy (yes/no/not the exact timing), on-going mental health difficulties (yes/no), previous mental health difficulties (yes/no), mode of birth preference if pregnancy medically uncomplicated (vaginal, cesarean section, do not know).

#### Fear of Birth Scale

The FOBS was used to measure participants FOC. FOBS consisted of two 100 mm Visual Analogue Scales [VASs] that were summed and then averaged. When completing the scale, participants answered: “How do you feel right now about the approaching birth?” and were instructed to place a mark on two scales (Scale 1 ranged from calm to worried and Scale 2 ranged from no fear to strong fear). The cut-off point ≥ 60 was used to measure severe FOC and < 60 as no, mild or moderate FOC (Hildingsson, Johansson, et al., 2014).

Part II consisted of items on received support, as well as facilitators and barriers in help-seeking for their FOC (14 items). In addition, there were three open questions with free-text responses. The first open question focused on support: “If no treatment is planned, would you like to receive treatment/support for your fear of childbirth?” If the participants answered “Yes,” they were asked to write in free text in the follow-up question: “If yes, please state why?” The other two open questions focused on their perceived barriers and facilitators to seeking support, including: “What obstacles have you experienced in seeking help for your fear of childbirth?” and “What would make it easier to seek help for fear of childbirth?”

### Survey Functionality

Survey functionality was tested by the research team (e.g., those who should see Part II were able to access it and vice versa). After small logic-errors and minor word changes were made, the survey was then pilot-tested on three expectant fathers. They provided feedback via email or phone, where only minor word changes were suggested to clarify some items.

### In-Depths Interviews

Fathers who self-identified as having an FOC when completing the survey were informed that they could email the lead researcher to participate in an in-depth interview. All five fathers who emailed the lead researcher were asked and agreed to participate in an in-depth interview. The interviews were conducted via telephone and were recorded. Interviews ranged in length between 48 and 131 min, with an average length of 78 minutes. The interviews were conducted using a semi-structured interview guide, focusing on their FOC.

### Data Analysis

#### Quantitative Data

First, we assessed if there were differences on fathers’ FOC based on whether they were expecting a child or currently had a child. Since there were no differences between these fathers based on their FOBS (see Table 1), their data were merged. We created several dichotomous variables to facilitate cross-tabulation. Relationship status was recoded into two groups: cohabiting with a partner (married or living with someone) and not cohabiting (single or with a partner, but not living together). Education was recoded into two groups: primary or high school education (9–13 years) and at least some college/university or higher. Country of birth was recoded as Sweden or other. Age was recoded into two groups: < 35 years and ≥ 35 years. Data analysis was performed in IBM SPSS Statistics for Windows 28.0 (IBM Corporation). Descriptive statistics were used to compute frequencies, percentages, mean, and standard deviation (*SD*). Chi-square test and Fisher’s exact test were used to assess differences in demographics and other covariates based on participants with FOBS < 60 and ≥ 60. Thresholds for significance were set at *p* < .05.

**Table 1.** Background Characteristics for the Study Participants in Relation to FOBS Score

Background characteristics	All expectant fathers *n* = 131 *n* (%)	FOBS< 60 *n* = 85 *n* (%)	FOBS ≥ 60 *n* = 44 *n* (%)	*p* value
Age (years; *N* = 130)				
< 35	63 (48.5)	42 (50)	21 (47.7)	.807
≥ 35	67 (51.5)	42 (50)	23 (52.3)	
Previous children (*N* = 121)				
Yes	51 (42.1)	31 (39.7)	20 (48.8)	.344
No	70 (57.9)	47 (60.3)	21 (51.2)	
Civil status (*N* = 131)				
Cohabiting with partner	128 (97.7)	83 (97.6)	43 (97.7)	1.000
Not cohabiting	3 (2.3)	2 (2.4)	1 (2.3)	
Highest level of education (*N* = 131)				
Primary or high school	50 (38.2)	29 (34.1)	21 (47.7)	.133
College/University	81 (61.8)	56 (65.9)	23 (52.3)	
Employment status^a^				
Employed	124 (94.7)	80 (94.1)	42 (95.5)	n.a.
Studying	4 (3.1)	3 (3.5)	1 (2.3)	
Parental leave	6 (4.6)	6 (7.1)	0 (0.0)	
Sick leave	1 (0.8)	0 (0.0)	1 (2.3)	
Sickness compensation and unable to work	0 (0.0)	0 (0.0)	0 (0.0)	
Unemployed	1 (0.8)	1 (1.2)	0 (0.0)	
Country of birth (*N* = 131)				
Sweden	119 (90.8)	78 (91.8)	39 (88.6)	.542
Other	12 (9.2)	7 (8.2)	5 (11.4)	
Place of residence (*N* = 131)				
City	89 (67.9)	57 (67.1)	30 (68.2)	n.a.
Town	24 (18.3)	14 (16.5)	10 (22.7)	
Village	18 (13.7)	14 (16.5)	4 (9.1)	
Partner pregnant (*N* = 131)				
Yes	90 (68.7)	59 (69.4)	30 (68.2)	.886
No	41 (31.3)	26 (30.6)	14 (31.8)	
Planned pregnancy (*N* = 90)				
Yes	40 (44.4)	24 (40.7)	16 (53.3)	n.a.
No	13 (14.4)	10 (16.9)	3 (10.0)	
Not the exact timing	37 (41.1)	25 (42.4)	11 (36.7)	
On-going mental health difficulties (*N* = 131)				
Yes	18 (13.7)	8 (9.4)	10 (22.7)	.039
No	113 (86.3)	77 (90.6)	34 (77.3)	
Previous mental health difficulties (*N* = 130)^a^				
Yes	47 (36.2)	31 (36.5)	16 (37.2)	.935
No	83 (63.8)	54 (63.5)	27 (62.8)	
Mode of birth preference if pregnancy medical uncomplicated (*N* = 89)				
Vaginal	69 (77.5)	46 (78.0)	23 (79.3)	n.a.
Cesarean section	6 (6.7)	3 (5.1)	3 (10.3)	
Do not know	14 (15.7)	10 (16.9)	3 (10.3)	

*Note.* Background information on participants collected between February and September 2022.

a Participants picked as many options as were applicable to them.

#### Qualitative Data

**Figure 1.**
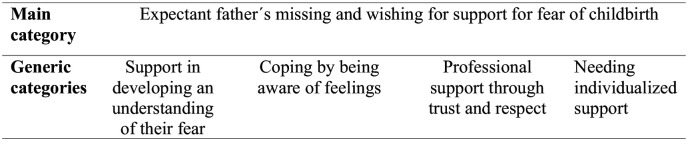
Four Generic Categories Explored in the Qualitative Data Analysis

A manifest content analysis approach (Elo & Kyngäs, 2008) was adopted to analyze responses to open survey questions informed by approaches adopted in other mixed-methods surveys (Hagström et al., 2022). Data about experiences of and preferences for FOC support, as well as fathers’ barriers and facilitators to seeking support were analyzed separately. Three steps were followed to analyze the text: (1) preparation, (2) organizing, and (3) reporting. The first author transcribed the interviews and the transcripts were merged with the free-text responses. After that, the data were read several times to facilitate an overall understanding of the content. Next, condensed meaning units were identified (e.g., text sharing a common meaning) and a performed line-by-line coding was carried out in a joint discussion between the first and the second authors. Subsequently, codes were sorted into four generic categories, describing one main category (Figure 1). The categories were agreed on by all authors. To strengthen the findings, quotations from the participants were added.

#### Data Integration

A contiguous approach to data integration was adopted with qualitative and quantitative findings reported separately (Fetters et al., 2013). In the quantitative data, we used descriptive and comparative analytic methods to describe the sample and examine participants’ experiences of and preferences for support, as well as participants’ barriers and facilitators to seeking support. Second, we analyzed the qualitative data to develop a deeper and more nuanced understanding of the participants’ preferences for and experiences of support, as well as their barriers and facilitators to seeking support.

## Results

In total, 131 participants answered Part I of the survey. Of those, 71 participants self-identified as having an FOC right now, and subsequently answered Part II. When considering the self-identified FOC item, three participants who marked that they did not have FOC had severe FOC according to FOBS; subsequently, they could not complete Part II. Of the 71 participants who completed Part II, 57% had FOBS ≥ 60, 13% had FOBS 50% to 59% and 30% had FOBS 0 to 49.

The participants had a mean age of 34.02 years (*SD* = 5.247) and most (90%) were Swedish-born, living with the mother of the child (97%), and had at least some university level of education (81%; Table 1). On the FOBS, participants scored a mean of 41.057 (*SD* = 29.649), where 34% of the 131 participants had a severe FOC (FOBS ≥ 60). In comparing those with and without a severe FOC against participants’ background characteristics, the only significant difference was that those with a severe FOC were more likely to report having on-going mental health difficulties (*p* = .039).

Of the five fathers who participated in in-depth interviews, one was expecting his first child, and the other four already had one child each, with three of them currently expecting their second child. All five participants have a severe FOC according to FOBS (≥ 60).

### Support for Severe FOC

In Part I, the vast majority (86%) of participants stated they received support from their birthing partner, friend, or another person, while nearly three quarters (71%) reported that they received partner support to a fairly or very large extent. There were no differences on received social support between those with and without an FOC.

In Part II, 40% of fathers with severe FOC wanted to receive support for their FOC (Table 2). However, 92% reported that no treatment was planned for receiving professional treatment. Of the three who received professional support, all reported that their received support helped them either to a lesser extent or not at all.

**Table 2.** Supportive Needs for Expectant Fathers When Experiencing Fear of Childbirth

Supportive needs	All fathers *n* = 71 *n* (%)	FOBS < 60 *n* = 31 *n* (%)	FOBS ≥ 60 *n* = 40 *n* (%)	*p* value
Would you like to receive support in relation to fear of childbirth? (*N* = 49)			*N* = 30	
Yes	16 (32.7)	4 (21.1)	12 (40.0)	
No	11 (22.4)	4 (21.1)	7 (23.3)	
Do not know	22 (44.9)	11 (57.9)	11 (36.7)	.290
Who would you like to deliver support for fear of childbirth?^a^				
Midwife	39 (54.9)	15 (48.4)	24 (60.0)	.329
Psychologist	23 (32.4)	9 (29.0)	14 (35.0)	.594
Physician	17 (23.9)	6 (19.4)	11 (27.5)	.425
Counselor	7 (9.9)	4 (12.9)	3 (7.5)	.449
Have you received professional treatment for fear of childbirth? (*N* = 63)				
Treatment is planned	0 (0.0)	0 (0.0)	0 (0.0)	n.a.
On-going treatment	0 (0.0)	0 (0.0)	0 (0.0)	
Completed treatment	3 (4.8)	0 (0.0)	3 (8.1)	
No treatment is planned	60 (95.2)	26(100.0)	34 (91.9)	
What kind of treatment is completed, on-going or planned?				
Counseling with midwife, physician, psychologist or counselor	2 (2.8)	0 (0.0)	2 (66.7)	
Treatment by CBT^b^, PDT^c^, PE^d^	1 (1.4)	0 (0.0)	1 (33.3)	n.a.
If the treatment was on-going or completed To what extent do you think your professional treatment has helped you?				
To a very large or large extent	0 (0.0)	0 (0.0)	0 (0.0)	
To a lesser extent or not at all	3 (100.0)	0 (0.0)	3 (100.0)	n.a.
If no treatment for childbirth fear is planned, would you like to have it? (*N* = 60)				
Yes	11 (18.3)	4 (15.4)	7 (20.6)	
No	25 (41.7)	15 (57.7)	10 (29.4)	n.a.
I do not know	24 (40.0)	7 (26.9)	17 (50.0)	
How important is talking about your fear of childbirth? (*N* = 56)				
It is very important/It is important	19 (33.9)	5 (21.7)	14 (42.4)	
It is not important at all	37 (66.1)	18 (78.3)	19 (57.6)	.108
Support design preference What types of support would you like to receive?^a^				
Dialogue with other parents	3 (4.2)	1 (3.2)	2 (5.0)	n.a.
Extra midwifery appointments	8 (11.3)	3 (9.7)	5 (12.5)	n.a.
Antenatal parental course	9 (12.7)	3 (9.7)	6 (15.0)	n.a.
Breathing and relaxation exercises	3 (4.2)	0 (0.0)	3 (7.5)	n.a.
The method of “Confident Birth”	7 (9.9)	2 (6.5)	5 (12.5)	n.a.
Mindfulness	3 (4.2)	1 (3.2)	2 (5.0)	n.a.
In what form could that support be provided?^a^ Individually for me	23 (32.4)	9 (29.0)	14 (35.0)	.594
Together with my partner	33 (46.5)	14 (45.2)	19 (47.5)	.845
In a group with other expectant fathers with FOC	7 (9.9)	3 (9.7)	4 (10.0)	n.a.
In a group with other expectant mothers and fathers with FOC	11 (15.5)	5 (16.1)	6 (15.0)	n.a.

a Several options available for participants.

b Cognitive behavioral therapy.

c Psychodynamic therapy.

d Psychoeducation.

One fifth (21%) of the participants with severe FOC wanted to receive professional treatment, while 50% did not know. Nearly, half (42%) stated that it was important or very important to talk about their FOC. The participants reported ambivalently regarding the types of support they would like to receive, as well as where the support would take place. However, over one-third (35%) wanted to receive support individually, 47.5% wanted support together with their partner, and 25% wished for group-based support.

As reported in Table 2, participants with severe FOC were more likely to want clinical support than those without FOC, where those with severe FOC wanted to receive support from a midwife (60%), a psychologist (14%), a physician (11%), and/or a counselor (3%). Nearly half (47.5%) of participants with severe FOC preferred to receive support with their partner. In considering where to receive support, 38% wanted physical meetings, 35% wanted individual support for them as expectant fathers, and 25% wanted support from internet links on pregnancy, childbirth, FOC and parenthood. Only three of 40 participants with severe FOC (8.1%) had an on-going or completed treatment for FOC. Treatment consisted of counseling with a midwife for two expectant fathers and cognitive behavioral therapy (CBT) for one expectant father. Of those who did not have a planned treatment, 20.6% wanted a treatment for FOC.

### Barriers and Facilitators in Help-Seeking

Fathers with severe FOC were more likely to report 1 or more barriers than those without FOC (Table 3). Of those with severe FOC, 80% stated that they had at least one barrier to seeking help, where 40% reported having one barrier, 27.5% had two barriers, and 12.5% had three or more barriers. Regarding fathers’ barriers, 20% of the participants with severe FOC had previous negative experiences of health care contacts and fear of not being listened to by others, respectively, while 40% stated they had unwanted social stigma surrounding their FOC and 12.5% had a fear that others would not believe they had FOC (Table 4).

**Table 3.** Associations Between FOC and the Number of Barriers a Father Reported Were Assessed

Total barriers *M* (*SD*)	All fathers *n* = 71 *n* (%)	FOBS < 60 *n* = 31 *n*(%)	FOBS ≥ 60 *n* = 40 *n* (%)	*p* value
	*M* = 1.17 *SD* = 1.13	*M* = .48 *SD* = .508	*M* =.80 *SD* =.405	n.a. .005
0	24 (33.8)	16 (51.6)	8 (20.0)	
1	23 (32.4)	7 (22.6)	16 (40.0)	
2	15 (21.1)	4 (12.9)	11 (27.5)	
3	7 (9.9)	3 (9.7)	4 (10.0)	
4	1 (1.4)	1 (3.2)	0 (0.0)	
5	1 (1.4)	0 (0.0)	1 (2.5)	
1 or more barrier	47 (66.2)	15 (48.4)	32 (80.0)	

**Table 4.** Prevalence of Reported Barriers Experienced in Seeking Help for Fear of Childbirth^a^

Barriers	All fathers *n* = 71 *n* (%)	FOBS < 60 *n* = 31 *n* (%)	FOBS ≥ 60 *n* = 40 *n* (%)	*p* value
Unwanted social stigma surrounding fear of childbirth				
Yes	24 (33.8)	8 (25.8)	16 (40.0)	
No	47 (66.2)	78 (91.8)	28 (63.6)	.210
Previous negative experience of health care contacts				
Yes	14 (19.7)	6 (19.4)	8 (20.0)	
No	57 (80.3)	79 (92.6)	36 (81.8)	.946
Fear of not being believed in by others				
Yes	7 (9.9)	2 (6.6)	5 (12.5)	
No	64 (90.1)	29 (93.5)	35 (87.5)	n.a
Fear of not being listened to by others				
Yes	12 (16.9)	4 (12.9)	8 (20.0)	
No	59 (83.1)	27 (87.1)	32 (80.0)	.429
Discomfort of having to face my own fears				
Yes	26 (36.6)	8 (25.8)	18 (45.0)	
No	45 (63.4)	23 (74.2)	22 (55.0)	.096

a Participants selected as many barriers as they wished.

The number of facilitators a father had regarding health-seeking behaviors did not differ between those with and without FOC (Table 5). Of those with severe FOC, 22.5% stated there were no facilitators to seeking support, while 45% reported one facilitator, 20% reported two facilitators, and 12.5% reported three or four facilitators. Of those with severe FOC, 25% reported that it would be a facilitator to receive professional support close to their home, 57.5% wanted easily available help, 17.5% wanted to choose different appointment times, and 30% wanted to receive professional support outside of normal working hours (Table 6).

**Table 5.** Number of Reported Facilitators

Reported facilitators	All fathers *n* = 71 *n* (%)	FOBS < 60 *n* = 31 *n* (%)	FOBS ≥ 60 *n* = 40 *n* (%)	*p* value
Average number of facilitators	*M* = 1.22 *SD* = 1.08	*M* = 1.13 *SD* = 1.08	*M* = 1.30 *SD* = 1.11	
0	20 (28.6)	11 (35.5)	9 (22.5)	n.a.
1	27 (38.6)	10 (32.3)	18 (45.0)	
2	13 (18.6)	5 (16.1)	8 (20.0)	
3	7 (10.0)	5 (16.1)	2 (5.0)	
4	3 (4.3)	0 (0.0)	3 (7.5)	

**Table 6.** Facilitators in Seeking Help for Expectant Fathers Experiencing Fear of Childbirth^a^

Facilitators	All fathers *n* = 71 *n* (%)	FOBS < 60 *n* = 31 *n* (%)	FOBS ≥ 60 *n* = 40 *n* (%)	*p* value
Receiving professional support close to my home				
Yes	16 (22.5)	6 (19.4)	10 (25.0)	.572
No	55 (77.5)	25 (80.6)	30 (75.0)	.962
Easily available help				
Yes	41 (57.7)	18 (58.1)	23 (57.5)	.878
No	30 (42.3)	17 (42.5)	17 (42.5)	.306
Being able to choose between different times for appointments				
Yes	12 (16.9)	5 (16.1)	7 (17.5)	
No	59 (83.1)	26 (83.9)	33 (82.5)	
Being able to receive professional support outside of standard working hours				
Yes	18 (25.4)	6 (19.4)	12 (30.0)	
No	53 (74.6)	25 (80.6)	28 (70.0)	

a Participants selected as many facilitators as they wished.

### Qualitative Results

#### Main Category: Expectant Father’s Missing and Wishing for Support for FOC

The main category composed four generic categories: (1) *support in developing an understanding of their fear*, (2) *coping by being aware of feelings*, (3) *professional support through trust and respect, and* (4) *needing individualized support* (Figure 1).

#### Support in Developing an Understanding of Their Fear

Participants reported that they did not receive support for their fear, including not being able to disclose their fear to their partner, not understanding what “fear of childbirth” meant for fathers, not being asked about their FOC or being offered further support by professionals on FOC.

As the pregnancy continued, participants found it difficult to comprehend that they would become fathers. They felt that it was more natural for their partners to know that they would be mothers due to physically carrying their child. As such, participants sometimes faced a mental barrier in seeking support.

You should not need to have a bad conscience if you do not feel like an expectant father at once. This is a big stigma thing. It takes time for the fathers to understand that they are fathers. **(P1)**

The participants repeatedly stated how they kept their fears and concerns about the pregnancy, as well as their partners’ and child’s health to themselves. As such, the participants felt like they could not be vulnerable and show their fears, but rather that they needed to show confidence and strength by pretending that they only had positive thoughts about the upcoming birth. Due to this line of reasoning, seeking support for fear became too complicated, as it would require them to admit that they had fears. The participants stated that expressing their true feelings in front of others, especially their partner, was seen as a difficult barrier to overcome. They further described a desire to break the stigma around FOC and that they would enjoy and benefit from discussing their FOC.

Yes, it would be a dream world if there is a bigger normalcy to talk about these fears . . . that would be wonderful, but I think that is a long way off. **(P5)**

Furthermore, the participants described that they had difficulty expressing and describing what their fears included.

[without my wife’s help] I would not have made contact [with a counsellor] because I would not have understood . . . you have to use more words than just fear of childbirth, when you describe it [fear of childbirth]. **(P4)**

Barriers in getting support for their FOC was that they were not offered any support even though it was needed.

Facilitating factors for receiving support for their FOC included being offered different types of information-based parenting preparation activities such as reading books, being active on social media sites for expectant parents, and using different mobile applications for expectant parents containing information on FOC. The participants gave examples of topics that were important to them including what could happen during pregnancy, after childbirth and the time after the birth of the child in terms of sleep, parenting and the relationship with their birthing partner.

Participants had a need to increase their understanding of their situation in becoming fathers to normalize their feelings about childbirth and fatherhood. They wanted to know how common it was for others in a similar position to have an FOC. Having this knowledge, participants stated, helped them feel like they were not alone in experiencing these feelings. Talking to other fathers who have fears was described as important to gain insight into the fact that others have similar feelings.

So all such activities [parenting preparation] I think still help you understand that something is going on. **(P2)**

Furthermore, the participants described that it was important to be able to talk to a midwife or other clinical professionals who have knowledge about FOC and to be able to ask questions when it comes to feelings and thoughts about becoming a parent, and to better understand what you are afraid of. Participants recognized their supportive role in helping their partner throughout the pregnancy period. However, they further noted that this could be difficult if they had a FOC, as constantly “remaining strong” for their partner and not sharing or working through their own emotions could lead to further anxiety and stress of not doing enough for their partner.

Tips on how I can support my partner . . . I would probably have felt a little better then. **(P1)**

Furthermore, it was easier for the participants to experience support if they had good role models growing up on how a father can be portrayed to feel safe before childbirth. Participants who had functioning and safe relationships with their own fathers tended to lean on their own fathers’ advice.

I have had a very good upbringing and have clear role models . . . I have got a pretty clear picture. **(P3)**

#### Coping by Being Aware of Feelings

Participants discussed the difficulty of not knowing how to cope with their fear or even if they were aware of their fearful feeling, so that, they could better process those feelings. They described self-medicating behaviors including not thinking about how they felt, trivializing their fear and avoiding showing their fear.

Participants reported that professionals did not offer them support. In addition, they did not feel like they could openly discuss their feelings with their partner. This left them feeling isolated, resulting in them suffering in silence, which consequently led to expressing stress and anxiety symptoms and feeling unprepared for becoming a father.

For a very long time I have felt that it was my fault for feeling the way I do, for feeling the way I do and having the feelings I have. **(P4)**

Participants expressed that they wanted to be aware of their feelings to better process their fear, which they believed helped them feel better and calmer. However, they stated that processing their fear could be pleasant but difficult. Participants described wanting to be the best support for their birthing partner, both during pregnancy and childbirth.

I want to be a support that she knows is there no matter what happens. She should not have to, not for a second, worry about how I feel or how I will react. She should know that I am stable, and I will fix the situation. **(P5)**

#### Trust and Respectful Professional Support

Participants talked about their need for professional support, especially with those familiar with FOC. They believed that it was important to have a trustful and respectful relationship with these clinical professionals, where they showed empathy to their own suffering.

The participants described that it was important for them to be treated respectfully by competent health care professionals. Being respectful included being invited and included in all prenatal care and childbirth matters. However, the participants reported that this respect was not always shown. Therefore, they wished to receive more professional support, where expertise was highly valued from midwives, psychologists, counselors and psychotherapy-trained professionals who would take time for them as individuals. Being included as an expectant father and as part of the prenatal process was helpful to them in understanding what was happening, which reduced their FOC.

Participants described a need for professionals to hold active discussions and lead the conversations. In doing so, participants stated that this would help them open up and express their feelings.

Participants wished to be included in care and desired that someone listened and cared about how they were doing. They appreciated being allowed to participate and wanted to receive straight and honest answers to their questions. They requested that health care professionals showed empathy, understanding and were sympathetic to their needs.

She asked how I was and how I felt. I felt an immense relief because for the first time someone sat down and listened. The visit to the labour ward provided some reassurance with the environment and gave the impression that they took my concerns seriously. **(P4)**

Participants experienced inadequate treatment by midwives in the form of feeling unwelcome and in the way. They had difficulties in getting opportunity to talk about their fears and they did not know where they could seek support for their fears.

You were a little bit in the way like a speck of dust in the corner of the delivery room. There are an incredible number of men who have that feeling. **(P5)**

By not being part of the care process and receiving support for their fear, participants described that the fear then remained within them. Participants described that it was important to feel trust in the care but that it was not always experienced.

#### Needing Individualized Support

Within this category, the participants talked about the importance of support customized for them, whether it is individual or in group as long as there was a focus on them as expectant fathers.

Participants expressed a need to be supported by a professional who takes the time to listen to them, the focus was on them as fathers, to feel and think and open up to new ways of seeing things and also to learn to manage their concern. Participants described that it was helpful to have early contact with the midwife and that the contact should be continuous to facilitate their ability to relax, open up and talk about their feelings and thoughts.

The dream had been to have the same midwife during the entire period! But in practical terms, I understand that it is a utopia. But the smaller the circle can be made, the calmer and safer I would have felt. At the moment, we don’t even know if we have the same midwife at MVC, [In English: Antenatal care clinic], or if we get the one who is available for the day. **(P1)**

Participants also wished that there was a structure around how it should be done when expectant fathers seek care and that it is necessary for health care professionals to book an appointment to them for a one-on-one counseling during pregnancy to ask direct questions, so that, there is possible to discover if they are carrying a fear and are in need of support. There was also a wish to get an appointment quickly and for it to be booked with little room to say no.

Like the dentist does. Here is a time for you! So you can cancel it if you can’t or don’t want to for that matter. That’s how I think it should be. **(P2)**

Participants described a great need to receive individually adapted support based on their own needs and that the question of support should be asked of them personally. It was important to be able to talk about one’s fears and raise problems in front of others to process what was difficult, and also taking part in and sharing each other’s experiences.

Some of the participants preferred individual counseling to get more attention, that the contact would feel like their own and that it can be difficult to talk in a group about tough feelings and situations. Distance-based counseling were also interesting to the participants in order to be able to remain anonymous.

Others preferred group discussions which could mean that they could share their own experiences and take part in the experiences and thoughts of others who were in the same situation. The participants wanted the groups to be small, so that, even shy personalities dare to speak up and that it should feel safe to open up.

## Discussion

The current mixed-method study on fathers found that one third (34%) had severe FOC and 40% of those wanted direct FOC support. The qualitative analysis revealed that participants found it difficult to disclose their FOC to their partner, despite the quantitative findings that three quarters had received partner support. In addition, participants with severe FOC had not received professional FOC support, and did not understand what “fear of childbirth” meant. Consequently, they did not know how to cope with their fear. As such, only 8.1% of participants with severe FOC received treatment, and the treatment was not perceived as helpful. Participants wanted more clinical support for their FOC, including individualized support, the chance to meet in groups with others with FOC, and to have their own clinical visits. However, they faced barriers to seeking support, including a discomfort in having to face their own fears, the stigma surrounding FOC, previous negative experiences of health care contacts, and fear of not being listened to. Facilitators to seeking support included easily available help, receiving professional support close to home, and being able to receive professional support outside standard working hours.

### Consequences of Expectant Fathers’ Mental Ill-Health

Expectant fathers are at increased risk of mental ill-health during the perinatal period, impacting children’s emotional and behavioral development (Wong et al., 2016). There seems to be bi-directional mental health risks, where expectant fathers’ depression, stress, and anxiety levels directly affect their FOC (Leach et al., 2016; Philpott et al., 2017), and their FOC directly affects their depression, stress, and anxiety (Ghaffari et al., 2022). This bi-directional relationship may stem from feelings of vulnerability (Greer et al., 2014), difficulties adjusting to becoming a father (Vallin et al., 2019), and lacking social and professional support (Moran et al., 2021; Vallin et al., 2019).

Our study demonstrates that severe FOC can be a heavy burden for fathers, including that they could not support their birthing partner due to heightened symptoms of anxiety and stress, making it difficult to adjust to fatherhood. They further described that they lived under gender roles that conditioned them to keep their feelings to themselves, convincing participants that they could not openly share their feelings. Fathers may not want to show their perceived weakness via their fears, as they do not want to upset or worry their pregnant partner (Moran et al., 2021). These social expectations make it difficult for fathers to talk about their fears, and thus, they suppress their own feelings (Eriksson et al., 2007). Participants reported that they felt alone by not being able to discuss their fear. Previous research highlights that by not being able to express their fear, fathers can develop further stress and anxiety (Philpott et al., 2019). The consequence to not expressing their fearful emotions can lead to self-medicating via avoidance behaviors, including non-participation in pregnancy and prenatal visits, working longer hours, withdrawal from social situations, and spending more time on social media (Melrose, 2010). The expectation to remain strong and composed may deter fathers from openly discussing their fears and concerns with their partners, health care providers, or support networks, further exacerbating feelings of isolation and distress. This might be an expression of that masculine identities are often sustained through men’s capacity to not ask for help, even if needed (Dolan, 2014). Health care providers and support organizations need to promote awareness about the stigma and barriers associated with FOC among fathers and create inclusive and non-judgmental environments that validate their experiences and encourage open communication about fears and emotions (Ruffell et al., 2019).

### Paternal FOC

There are counseling programs and support efforts for women, but not men, with FOC in Sweden (Svensk förening för Obstetrik och Gynekologi [SFOG], 2017). Men want support from health care professionals, but similar to the current findings, those with FOC tend not to talk about their fears, making it difficult for health care professionals to identify them (O’Brien et al., 2017). Using validated instruments, such as FOBS can be helpful in identifying fathers with FOC. In our study, most fathers who had a self-described FOC also had a severe FOC according to FOBS. However, a minority of fathers did not think they had an FOC even though they scored ≥ 60 on FOBS. In addition, there have been different cut-offs used for severe FOC, including 50 or higher (Haines et al., 2011; Hildingsson, Johansson, et al., 2014). If this cut-off was used in the current study, then 89% of fathers who had a self-described FOC also had FOC according to FOBS. Nevertheless, FOBS was initially developed for women and the accuracy and comprehensiveness of this screening tool for evaluating fathers’ FOC requires further investigation, including if new paternal scales should be used (Ghaffari, Elyasi, et al., 2021; Guo et al., 2023). The current study found that since fathers did not always recognize what FOC meant, using a variety of descriptive language and scenarios might be helpful for fathers to understand and express their emotions; thus, recognizing their FOC.

### Social and Professional Support

Social support theory suggests that individuals derive emotional and psychological benefits from their social relationships, including interactions with health care professionals like midwives (Shumaker & Brownell, 1984). By attending prenatal visits, parental education groups, and learning practical skills, they can feel more connected, fostering a sense of control and reducing their fears (Li et al., 2009). In the current study, most participants with severe FOC (82%) reported that they received social support, such as from their partner, friends, and family. This contrasts with Eriksson et al.’s finding that 65% received no support (Eriksson et al., 2005). When fathers receive social support, their feelings are validated, as this allows them to have a safe space to express their concerns (Bergström et al., 2013; Ghaffari et al., 2022) Receiving this emotional validation helps them cope with their fears (Bergström et al., 2013; Ghaffari et al., 2022). However, in the interviews with the participants, fathers stated that it can be difficult to discuss their feelings with their partner. This might imply that even though fathers receive support from their partner, they actually do not tell their partner the full extent of their suffering. Future research should further explore the concept of partner support for fathers with severe FOC.

Those with severe FOC wanted to receive professional support, including from midwives, psychologists, physicians, and counselors. Fathers value midwife support (Greer et al., 2014; Hildingsson, Johansson, et al., 2014), and once received, they tend to feel more at ease and secure (Johansson et al., 2015). When midwives address fathers’ specific fears, answer their questions, and provide evidence-informed information, their FOC decreases. Current participants believed midwives were competent professionals, but had less knowledge around paternal mental health issues and how to specifically support them. For example, fathers are not seen as clients, have no medical records, and thus are not routinely screened for any mental health issue, including FOC within Swedish antenatal care (SFOG, 2017). Therefore, while there is research being completed on better measuring paternal FOC (Calpbinici et al., 2023; Ghaffari et al., 2020; Guo et al., 2023; Hildingsson, Johansson, et al., 2014), routine screening, diagnosing and treating fathers will not happen, unless they are considered part of the prenatal care system.

Several international and national organizations state that midwives are tasked with supporting the health of the whole family, including the father (International Confederation of Midwives [ICM], 2014; SFOG, 2017; Swedish Association of Midwives, 2018). The current research adds to what several literature reviews on expectant fathers have already stated: that fathers are marginalized or completely left out of care (Moran et al., 2021), leading to worsened mental health (Ghaffari, Elyasi, et al., 2021; Philpott et al., 2019), which both directly and indirectly impacts their partner’s and child’s outcomes (Ramchandani et al., 2005).

However, many midwives operate within traditional health care settings where support is primarily focused on expectant mothers (SFOG, 2017). As such, fathers can feel isolated, invisible, marginalized, and unsupported in prenatal, perinatal, and postnatal care (Venning et al, 2021; Wells, 2016), contributing to lasting fears (Jungmarker et al., 2010). In addition, they may perceive their role as secondary to expectant mothers and therefore be reluctant to express a need for support (Darwin et al., 2017). As such, midwives and other clinical professionals working with FOC might need to take proactive approaches to supporting fathers and creating a safe and trustful environment for them to openly express their feelings. To offer more emotional support and create a safe space for fathers to open up about their feelings, midwives can employ several strategies including actively listening to fathers’ concerns in a non-judgmental attitude, validating their feelings, and demonstrating empathy without creating stigma for the FOC. Providing opportunities for fathers to speak individually with the midwife, individual consultations, making the expectant fathers more willing to talk about sensitive emotions and experiences than in front of their partner. Previous research highlights that professional support increases fathers’ involvement and strengthens them in supporting their partner during childbirth (Anderson & Gill, 2014; Sioma Markowska et al., 2016; Sengane & Nolte, 2012).

In line with current findings, as well as with literature reviews on paternal FOC (Moran et al., 2021), advocacy efforts aimed at policy changes within health care systems may also be necessary to promote greater accessibility and inclusivity for fathers. Fathers could be more included by prioritizing paternal involvement in prenatal care, advocating for expanded support services tailored to their individual needs, and challenge existing norms and practices that may exclude or marginalize them from the perinatal care experience.

The current results further illustrate that nearly one quarter of fathers with severe FOC would like to attend support groups with others. Peer support groups can allow fathers to share experiences, exchange tips and normalize their feelings (O’Brien et al., 2016). In turn, this promotes friendships and reduces isolation (Hanna, 2018). Since many fathers reported feeling isolated and alone, having opportunities for them to attend FOC support groups could be helpful in reducing their fears. Some of the participants also wanted to receive distance-based counseling where they could remain anonymous. This preference can be understood within the context of societal norms that discourage men from openly discussing emotional struggles or seeking help (Eriksson et al., 2007). Distance-based counseling offers a level of anonymity that may appeal to expectant fathers who feel reluctant to seek face-to-face support due to concerns about judgment or stigma (Bergström et al., 2013; Eriksson et al., 2007; Moran et al., 2021).

### Strengths and Limitations

This is one of the first mixed-method studies examining fathers’ support needs, as well as their barriers and facilitators to seeking support. We further compared differences of support based on fathers’ FOC, as well as how and why they experienced FOC. However, a limitation is that the sample size is relatively small, as only those who self-reported having an FOC could answer the items on facilitators and barriers to seeking support. Previous research further highlights that recruitment of fathers is difficult. In fact, the larger project this study is embedded in used the same recruitment techniques for expectant mothers, where there were 1406 participants, suggesting that other recruitment methods should be tested to better encourage fathers to complete FOC surveys.

A potential strength is that the current study used data from both expectant fathers and those who already are fathers. This helps to highlight that FOC is not only a concern during pregnancy, but also can persist after birth too. However, some variables had 30% missing data, which might impact their generalizability. Items with high missing data included: (a) their mode of birth preference if pregnancy was medically uncomplicated (vaginal/cesarean section/do not know), (2) would you like to receive support in relation to FOC (yes/no/do not know)?, and (3) how important is talking about your FOC (it is very important/it is important/it is not important at all)? For the first item, it might be that fathers perceived a medically complicated birth and so skipped the item. However, it is unclear why the latter two items were skipped. Further investigation might be warranted regarding what fathers are and are not willing to share regarding their FOC.

Another strength is that participants for this study were recruited through three disparate recruitment techniques, allowing for participants to represent all counties in Sweden. However, when comparing our study to other national studies in Sweden (Eriksson et al., 2005), the current study found more participants with FOC. This might be because the paid Facebook advertisements specifically asked potential participants to complete the survey if they felt they had FOC. One first time-father and four current fathers (three expecting their second child, and one who had a child) participated in a semi-structured interview. While five interviews is not a large qualitative study, the interviews were in-depth, lasting 74 min on average. In addition, the data from the three open-ended survey items from the quantitative survey on all fathers with FOC further added to the qualitative analysis, lending important insights into the experiences of fathers with FOC, their needs, and perceived barriers and facilitators to support.

## Conclusion

This study found that one third of fathers had severe FOC and wanted support for their fear. However, unlike expectant mothers with FOC, fathers lacked support from prenatal health services. Paternal FOC is a burden and is associated with further emotional distress that they must handle themselves. Fathers reported that they did not want to disclose their FOC, as this might worry their birthing partner. In addition, they faced gender norms around masculinity, resulting in staying silent on their FOC. These findings highlight the importance of clinical professionals asking fathers about their feelings during their birthing partner’s pregnancy and offering them their own appointment with the midwife to investigate further needs of support. By addressing policy changes that prioritize the needs of fathers, health care providers and policymakers can work toward creating a more inclusive and supportive environment for all parents during the perinatal period.
